# Association between Diffusion Tensor Imaging, inflammation and immunological alterations in unipolar and bipolar depression: a review

**DOI:** 10.1192/j.eurpsy.2023.1261

**Published:** 2023-07-19

**Authors:** R. Aronica, P. Enrico, P. Brambilla, G. Delvecchio

**Affiliations:** 1 Department of Pathophysiology and Transplantation, University of Milan, 20122 Milan; 2 Department of Neurosciences and Mental Health, Fondazione IRCCS Ca’ Granda-Ospedale Maggiore Policlinico, Milan, Italy

## Abstract

**Introduction:**

Major Depressive Disorder (MDD) and Bipolar Disorder Depression (BDD) are common psychiatric illnesses characterized by structural and functional brain alterations and signs of neuroinflammation. In line with the neuroinflammatory pathogenesis of depressive syndromes (Mechawar N, Savitz J. Neuropathology of mood disorders: do we see the stigmata of inflammation? Transl Psychiatry. 2016;6(11):e946), recent studies have demonstrated how white matter (WM) microstructural impairments detected by Diffusion Tensor Imaging (DTI) are correlated to peripheral immunomarkers in depressed patients.

**Objectives:**

In this context, the aim of our review is to report an updated overview of the evidence on the correlation between the blood immuno-markers changes and the brain WM disruptions in MDD and BDD patients.

**Methods:**

Based on PRISMA 2020 guidelines (Page MJ, McKenzie JE, Bossuyt PM, Boutron I, Hoffmann TC, Mulrow CD, et al. The PRISMA 2020 statement: An updated guideline for reporting systematic reviews. BMJ. 2021;372), we performed a systematic search on original DTI studies exploring the association between WM integrity, neuroimmune alterations and inflammation in patients affected by MDD or BDD.

**Results:**

Concerning MDD, most of the reviewed studies provided evidence of a link between systemic immune dysregulation, detected through the elevation of peripheral markers (IL-1β and TNF-alfa) or an altered ratio between proinflammatory and counterregulatory cytokines (IL-8/IL-10), and DTI alterations in specific WM tracts, such as the genu of corpus callosum and the IFOF. As for the BDD, we detected an increase of pro-inflammatory molecules (such as TNF-alfa, IL-8, IFN-γ etc.) that correlated with DTI changes in different cerebral areas such as cingulum, forceps, corona radiata, corpus callosum, longitudinal fasciculus and internal capsule. Furthermore, other molecules seem to play a specific role in BDD pathogenesis, including counter-regulatory cytokines, kynurenine and specific lymphocyte classes, such as Th1 and Th17.

**Conclusions:**

Taken together, these pathogenetic insights could outline an integrated clinical perspective to affective disorders, helping psychiatrists to develop novel biotype-to-phenotype models of depression and opening the way to tailored approaches in treatments.

**Disclosure of Interest:**

None Declared

